# Fluoride Intake Through Dental Care Products: A Systematic Review

**DOI:** 10.3389/froh.2022.916372

**Published:** 2022-06-10

**Authors:** Hanan Saad, Raphaëlle Escoube, Sylvie Babajko, Sophia Houari

**Affiliations:** ^1^Laboratory of Molecular Oral Physiopathology, Centre de Recherche des Cordeliers, INSERM, Université Paris Cité, Sorbonne Université, Paris, France; ^2^AP-HP, Dental Medicine Department, Pitié-Salpétrière Hospital, GHN-Université Paris Cité, Paris, France; ^3^Laboratoire de Géologie de Lyon, UM R5276, CNRS, Université Lyon 1, École Normale Supérieure de Lyon 46, Lyon, France

**Keywords:** fluoride, drinking water, diet, toothpaste, dental products, urine

## Abstract

Fluoride (F) is added to many dental care products as well as in drinking water to prevent dental decay. However, recent data associating exposure to F with some developmental defects with consequences in many organs raise concerns about its daily use for dental care. This systematic review aimed to evaluate the contribution of dental care products with regard to overall F intake through drinking water and diet with measurements of F excretion in urine used as a suitable biomarker. According to the Preferred Reporting Items for Systematic Reviews and Meta-Analyses (PRISMA) guidelines using keywords related to chronic exposure to F in the human population with measurements of F levels in body fluids, 1,273 papers published between 1995 and 2021 were screened, and 28 papers were finally included for data extraction concerning daily F intake. The contribution of dental care products, essentially by toothbrushing with kinds of toothpaste containing F, was 38% in the mean regardless of the F concentrations in drinking water. There was no correlation between F intake through toothpaste and age, nor with F levels in water ranging from 0.3 to 1.5 mg/L. There was no correlation between F intake and urinary F excretion levels despite an increase in its content in urine within hours following exposure to dental care products (toothpastes, varnishes, or other dental care products). The consequences of exposure to F on health are discussed in the recent context of its suspected toxicity reported in the literature. The conclusions of the review aim to provide objective messages to patients and dental professionals worried about the use of F-containing materials or products to prevent initial caries or hypomineralized enamel lesions, especially for young children.

## Introduction

Fluoride (F) is the lighter halogen element and is largely present in food and drinking water with levels depending on the geological environment of the area. It is also added to dental care products used for oral hygiene and dentistry to prevent dental decay. It is admitted that tooth brushing with fluoridated toothpaste is a fundamental cornerstone for the prevention of early childhood caries [1]. It protects against caries by generating fluoridated apatite more resistant to acids produced by oral bacteria, increasing the remineralization process, and inhibiting bacterial enolase activity [2, 3]. However, limits to the prescription of F have been repeatedly advised, mostly because of the narrow safety range for its use. According to the European Food and Safety Authority (EFSA), the recommended doses to prevent caries have been evaluated approximately 0.05–0.07 mg/kg/day, which is close to the amount that may cause enamel hypomineralization, called dental fluorosis (>0.1 mg/kg/day) [4].

The main sources of F intake are fluoridated drinking water, dietary F, infant formulas, and F-containing dental care products, especially toothpaste. Some foods and beverages contain high levels of F, such as tea [5]. The increased prevalence of dental fluorosis indicates that some young children are exposed to F from sources other than drinking water, essentially the F-containing toothpaste they may swallow. F can substitute hydroxyl of the hydroxyapatite containing matrices, to form fluorapatite, underlying its extracellular effects in enamel, dentin, and bone [6]. F tropism for apatite explains its expected reinforced effects on enamel as well as dental and bone fluorosis when absorbed in excess [7]. Besides biomineralized matrices, many experimental studies report F effects on cell differentiation, proliferation, and apoptosis that may explain its toxic effects on the development and the physiology of many other tissues and organs when ingested at high doses [8–11]. The severity of F effects is related to the dose and duration of exposure as well as to its combination with other environmental factors as suggested by experimental studies on rodents and zebrafish [7, 12, 13]. The severity of F effects also appears to be contingent on the genetic background in rodents and humans and renal function [8, 14–17]. Once absorbed, F travels throughout the body *via* the blood circulation before being filtered by the kidney and excreted in urine, which thus ensures the majority of F removal from the body. Approximately 60% of ingested F by healthy adults are excreted in the urine, but only 45% for children, with the rest re-circulating into the plasma or deposited into the bone [18]. As a consequence, plasma and urinary excretion reflect a physiologic homeostasis determined by previous F intake, rate of F uptake and removal from bone, and the efficiency with which the kidneys excrete F.

Dental fluorosis and other F side effects on health may occur due to F overload from a combination of various sources, such as drinking water, dental care products used for caries prevention, medication with fluoridated products, and anesthetics, each source being innocent alone but with an unclear dose-response relationship when combined [19]. Due to the general awareness of relations between human environment and health, many patients are currently questioning their physicians and dentists about the safety of the prescribed treatments. Dentists strongly advice to brush teeth at least two times a day with fluorinated toothpastes, preconize fluorinated varnishes to protect children's teeth from caries, and higher fluorinated gels for specific patients and use biomaterials, such as adhesives or ionomer cements, for conservative dentistry and orthodontic treatments that may contain F.

The aim of this study is to provide a qualitative and descriptive analysis of the numerical data to evaluate the contribution of dental care products in the total daily fluoride intake (TDFI) based on urine monitoring and regarding the literature from 1995 to 2021. In the light of these results, dentists will be able to qualify the place that F takes in prevention and treatment programs in the overall systemic exposure of patients.

## Methods

**Figure 1:**
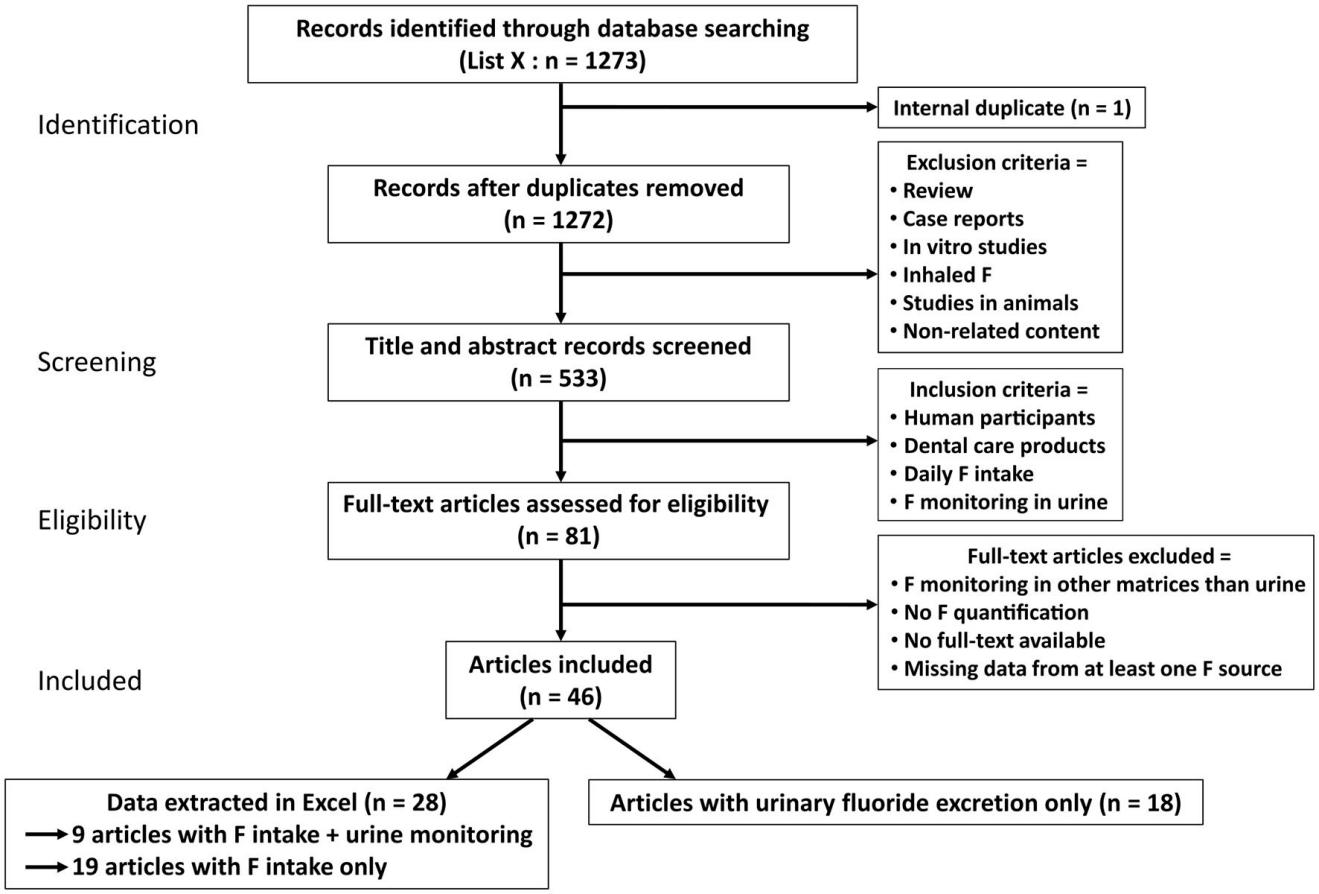
The Preferred Reporting Items for Systematic Reviews and Meta-Analyses (PRISMA) flowchart for the systematic review. From the 1,273 articles found in PubMed included in the search, 46 studies were included and 28 selected in this review for their analyses. Among the 28 articles, 19 only listed the estimated total daily fluoride intake (TDFI) (Table 1). The other nine articles had information regarding both the TDFI and the daily urinary fluoride excretion (DUFE) (Table 2).

This systematic review is conducted according to the Preferred Reporting Items for Systematic Reviews and Meta-Analyses (PRISMA) guidelines (Figure 1).

**Table 1:** Number of participants, their mean age and country of residence, with the associated F concentration in tap water (mg/L) in articles with only estimation of F intake in our Excel database.

**Order of publications**	**Number of subjects**	Number of toothpaste users*	**Mean age (year)**	**Country**	**F concentration in tap water (mg/L)**
Levy et al. [20]_low water	75	11	0.8	USA	0.3–0.6
Rojas-Sanchez et al. [21]_low water 1	11		2.4	USA	0.3
Rojas-Sanchez et al. [21]_low water 2	14		2.3	USA	0.3
Rojas-Sanchez et al. [21]_high water	29		2.3	USA	1
Levy et al. [22]_poor to high water, 20 months	615		1.7	USA	0.3–2
Levy et al. [22]_poor to high water, 28 months	552		2.3	USA	0.3–2
Levy et al. [22]_poor to high water, 36 months	506		3.0	USA	0.3–2
Murakami et al. [23]_poor water	93		4.1	Japan	0.12
Levy et al. [24]_poor to high water, 36-72 months	785		4.5	USA	0.1–3.1
Martinez-Mier et al. [25]_poor water 1	21	19	2.6	Mexico	0.04
Martinez-Mier et al. [25]_poor water 2	21	20	2.5	Mexico	0.07
Paiva et al. [26]_medium water 1	32		2.1	Brazil	0.7
Paiva et al. [26]_medium water 2	39		2.4	Brazil	0.7
Pessan et al. [27]_medium water, 4-5 years	10	9	4.5	Brazil	0.7
Pessan et al. [27]_medium water, 6-7 years	11	10	6.5	Brazil	0.7
Cardoso et al. [28]_low water, adult	5		30.0	Brazil	0.3
Cardoso et al. [28]_medium water 1, adult	5		30.0	Brazil	0.7
Cardoso et al. [28]_medium water 2, adult	5		30.0	Brazil	0.7
Omena et al. [29]_high water	58		2.4	Brazil	0.94
de Almeida et al. [30]_medium water	33		27.0	Brazil	0.7
Miziara et al. [31]_medium water	379		4.0	Brazil	0.7
Levy et al. [32]_high water, with fluorosis	367		9.0	USA	0.9
Levy et al. [32]_high water, no fluorosis	163		9.0	USA	1
Lima-Arsati et al. [33]_medium water	23		2.3	Brazil	0.72
Amaral et al. [34]_poor water, toothpaste 1	NA		1.5	Brazil	0.204
Amaral et al. [34]_poor water, toothpaste 2	NA		1.5	Brazil	0.213
Amaral et al. [34]_poor water, toothpaste 3	NA		1.5	Brazil	0.247
Zohoori et al. [35]_poor water	3		10.2	UK	0.19
Zohoori et al. [35]_high water	2		1.0	UK	0.97
Abuhaloob et al. [36]_poor water	81	2	4.0	Palestine	0.21
Abuhaloob et al. [36]_high water 1	72	5	4.1	Palestine	0.91
Abuhaloob et al. [36]_high water 2	63	4	4.1	Palestine	1.71
Lima et al. [37]_medium water	67		4.2	Brazil	0.64
Oliveira et al. [38]_medium water	58		4.2	Brazil	0.6

*Number of toothpaste users if different from the total number of participants.

**Table 2:** Number of participants, their mean age and country of residence, with the associated F concentration in tap water (mg/L) in articles regarding estimated F intake and urine monitoring in our Excel database.

**Order of publication**	**Number of subjects**	Number of toothpaste users*	**Mean age (year)**	**Country**	**F concentration in tap water (mg/L)**
Villa et al. [39]_medium water	20		4.4	Chile	0.58
Zohoori and Rugg-Gunn [40]_low water	32	30	4	Iran	0.32
Haftenberger et al. [41]_poor water	11	10	4.2	Germany	0.25
Pessan et al. [42]_medium water, carie-free	9		5.1	Brazil	0.59
Pessan et al. [42]_medium water, carie	11		5.4	Brazil	0.59
Maguire et al. [43]_poor water	18		6.9	UK	0.08
Maguire et al. [43]_low water	8		6.9	UK	0.47
Maguire et al. [43]_high water	3		6.9	UK	0.82
Zohoori et al. [44]_poor water	9		2.1	Brazil	0.04
Zohoori et al. [44] _medium water	5		3.2	Brazil	0.64
Zohoori et al. [45]_low water	21		6.8	England	0.3
Zohoori et al. [45]_high water	12		6.6	England	1.06
Idowu et al. [46]_poor water, child	32		4.4	US	0.04
Idowu et al. [46]_high water, child	29		4.4	US	3.05
Idowu et al. [47]_poor water, adult	31		33.1	Nigeria	0.04
Idowu et al. [47]_high water, adult	29		34.6	Nigeria	3.05

*Number of toothpaste users if different from the total number of participants.

### Search Strategy

The following search equation was entered in PubMed/Medline using the Booleans: (((dent^*^) OR (mouth^*^) OR (teeth) OR (tooth^*^) OR (enamel)) OR ((resin?) OR (“glass ionomer^*^”) OR (“bioactive glass^*^”) OR (composite?)) AND ((urin^*^ fluori^*^) OR (plasma^*^ fluori^*^) OR (“blood fluori^*^”) OR (“saliva fluori^*^”) OR (“bone fluori^*^”) OR (“hair fluori^*^”) OR (“nail fluori^*^”)) AND ((1995/1/1:2021/12/31[pdat]) AND (english[Filter] OR french[Filter]))) OR (((dent^*^) OR (mouth^*^) OR (teeth) OR (tooth^*^) OR (enamel)) OR ((resin?) OR (“glass ionomer^*^”) OR (“bioactive glass^*^”) OR (composite?)) AND ((“chronic fluoride”) OR (“chronic exposure to fluoride”) OR (“chronic fluoride exposure”) OR (“fluoride intake”) OR (“daily fluoride intake”) OR (“systemic fluoride”)) AND ((1995/1/1:2021/12/31[pdat]) AND (english[Filter] OR french [Filter]))).

Open access articles were retrieved and those with restricted access were retrieved through institutional access. Only two articles were excluded because the full-text was not accessible.

We checked that none of the included studies in this review were retracted due to error or fraud.

### Eligibility Criteria

#### Inclusion Criteria

When establishing the search equation, language was limited to English and French, and articles were restricted from 01/01/1995 to 31/12/2021. The articles were selected taking into account the following inclusion criteria: (1) studies with human participants, (2) studies involving topical use of F-containing dental care products, (3) studies estimating the TDFI from water, beverages, such as juices, milk and infant formulas, meals, and dental care products which are mainly toothpastes in this review, and (4) studies monitoring F exposure through urine as a contemporary biomarker.

#### Exclusion Criteria

The exclusion process consisted of two steps. The first was applied before the inclusion of articles with the following criteria on title and abstract: (1) studies conducted on animals, (2) studies *in vitro*, (3) articles focusing on inhaled F, which may be found in some anesthesia, (4) articles with no related content to F exposure, and (5) reviews and case reports. The second step consisted on excluding those that had the following criteria: (1) F monitoring in other matrices than urine (plasma, saliva, nails, and hair), (2) articles with no F quantification or estimation, (3) articles that were not accessible, and (4) studies with missing data from at least one source of F either from water, beverages, solid food, or dental care product.

### Process of Study Selection

First, all articles resulting of the search equation were entered in Zotero software. Elimination of duplicates was performed. Then the screening of title and abstract by two independent reviewers (HS et SH) was carried out according to the first step of the exclusion process. The same reviewers proceeded to select the articles by applying the inclusion criteria. The content of the abstract of each study was analyzed and the articles with relevant information regarding the subject of the current review were carefully chosen. Finally, the selected articles were evaluated through full-text analysis to determine which of them would be useful for the elaboration of the systematic review. This second step of the exclusion process was performed independently and in duplicate by each reviewer to compare the recorded information and correct the differences that were found during this step. In the case of disagreement between reviewers, a third reviewer (SB) was involved to resolve it.

### Data Charting Process

Among the articles included, those relating the daily F intake of each source and monitoring the F in urine were selected. All data were entered in Excel software and was sorted to identify the authors, year of publication, title of article, country of study, number of participants, age and gender of participants, sources of exposure to fluorides, F concentration in tap water, and F monitoring in urine. Data extraction was performed by HS and RE.

### Data Synthesis

Studies' characteristics are as follows:

- Year: from 1995 to 2021. The year 1995 was selected because it was the last recent date mentioned by Fejerskov et al. [48], which compiled all data from the previous years.
- Country: Supplementary Figure 1 represents the Mondial geographical repartition of the included articles in Excel. The percentage was calculated by counting the number of studies conducted in a country and dividing it by the total number of articles in Excel.
- Age: it was notified when it was presented (Tables 1, 2).
- Size of the cohort: the number of subjects was reported for each study when available. Moreover, the number of participants using dental care products was mentioned when it differed from the total number of participants (Tables 1, 2).
- Gender: proportion of men or women in participants was reported when data were available.
- All the articles selected in Excel are listed in Table 1 for those estimating F intake and in Table 2 for those estimating F intake and urine monitoring with the number of subjects, their mean age, countries of residence, and F concentration in tap water.

For outcome measures, we extracted information for the following parameters when they were available:

- The concentration of F in tap water in the area was entered in Excel. From our database, a range of F concentration was determined and quartiles were calculated. We obtained different categories of water depending on F content: poor (<0.3 mg/L), low (0.3–0.51 mg/L), medium (0.52–0.77 mg/L), and high (0.77–1.5 mg/L).
- Estimated intakes of F sources were water, beverages, diet, dental products, and supplements. Depending on the authors, the report of the sources may vary by combination of the listed sources.
- Method of assessment of daily dietary F intake (DDFI): diet diary during 2–3 days, duplicate plate method, diet history, and food frequency questionnaire (FFQ).
- Method of assessment of F intake from dental products: sample collection, toothpaste applied/expectorate collected, toothbrushing questionnaire, and toothpaste/toothbrush weighing before and after brushing.
- Contribution of dental care products to the TDFI: some values were easily found in articles and others needed to be calculated, when possible, by taking into account that not all participants had oral hygiene habits.
- Fluoride excretion: urine, or urine and feces (due to infant participants wearing diapers).
- Kinetic studies: those studies were based on timeline variations of F exposure depending on the dental care uses.
- Method of assessment of F in urine: urinary F concentration, urinary F excretion (by collecting 24-h urine, or spot urine, or time-controlled urine). Daily urinary fluoride excretion (DUFE) or F retention were reported when presented in the article.
- Analytical method: F-ion selective electrode, hexamethyl-di-siloxane diffusion, or not reported.
- Validity of data and methods: F intake, F excretion (urine collection), and F analytical method, or not reported.
- Reporting of the investigation of any relationship between F-containing dental products and F excretion.

### Data Reporting

All data were reported and homogenized for intercomparisons in μg/day or μg/kg bw/day for F intake from diet and toothpaste, TDFI and DUFE, and in mg/L for F concentration in tap water.

**Figure 2:**
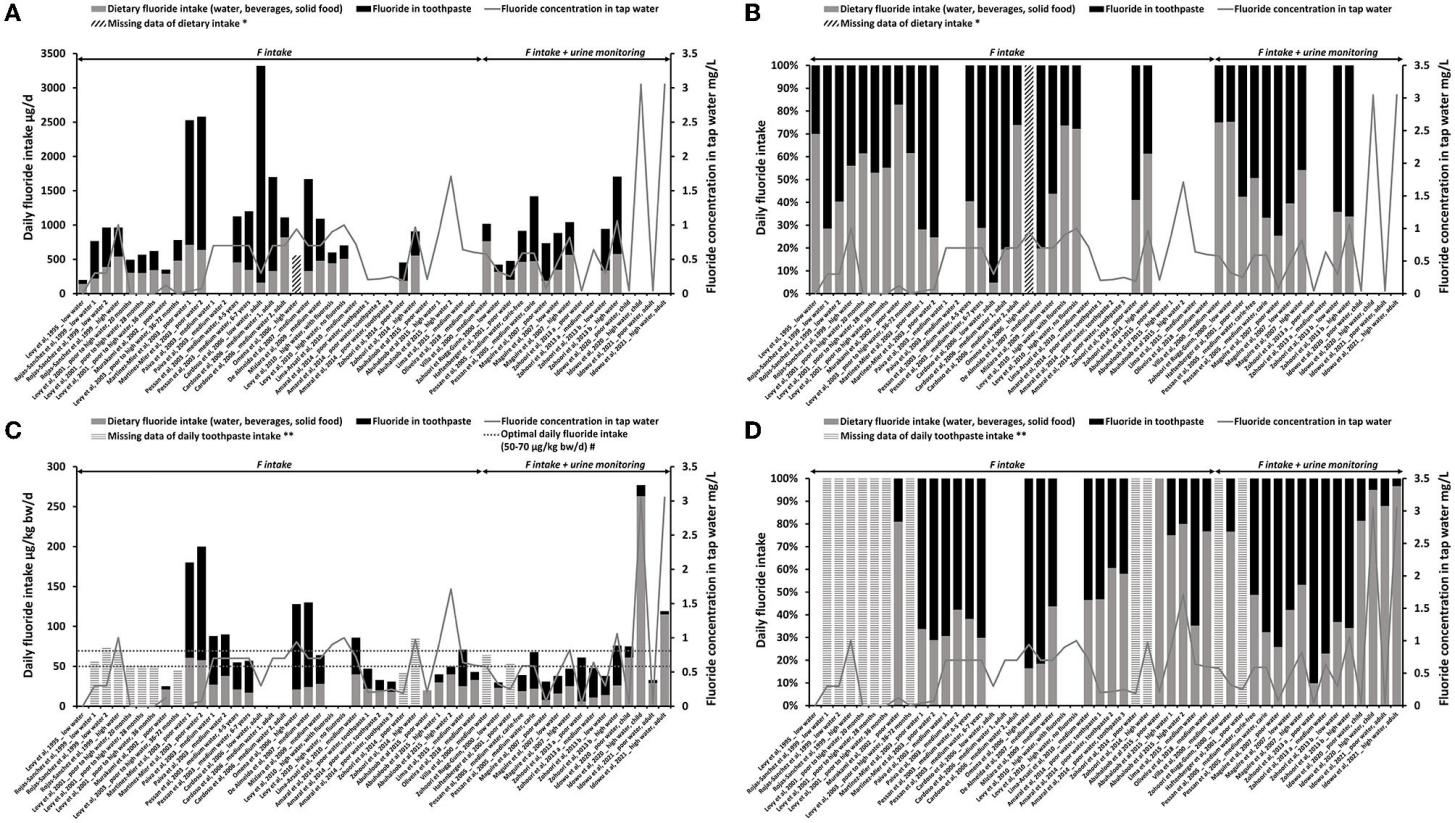
Estimated F intake from diet (water, beverages, and solid foods) (gray bars) and from toothpaste (black bars). F concentration in tap water is represented by the gray line (mg/L). **(A)** Total daily fluoride intake with dietary and toothpaste inputs (μg/day). **(B)** The contribution of daily diet (water, beverages, and solid foods) and toothpaste (%) to the estimated F intake in μg/day [based on **(A)**]. **(C)** TDFI with dietary and toothpaste inputs (μg/kg bw/day) with reference values of optimal daily F intake [50–70 μg/kg bw/day, the European Food and Safety Authority (EFSA)]. **(D)** The contribution of daily diet (water, beverages, and solid foods) and toothpaste (%) to the estimated F intake in μg/kg bw/day [based on **(C)**]. *Diagonal hatched bars represent missing data of daily dietary F intake (DDFI). **Horizontal hatched bars represent missing data of daily F intake from toothpaste. ^#^Optimal range of daily F intake reported in the literature.

In some publications, the contribution of diet and toothpaste was not reported. To be able to compare all the selected published data, we calculated the percentage of contribution of diet and toothpaste to the mean TDFI. This was based on the mean F intake extracted from the included articles regardless of the availability of percentage data. Despite not being optimal, this allowed to keep the maximum number of articles (Figure 2).

## Results

From our initial selection of 1,273 articles, 46 met the inclusion criteria and only 28 were included in our systematic review as digital data concerning the estimation of daily F intake was reported by authors (Figure 1). Among these 46 articles, 18 were used only for the discussion and were not included into our database since they reported urine F excretion without any estimation of the TDFI [49–66]. Concerning the 28 selected studies, they were carried out in countries all over the world with almost half of the studies (43%) carried out in Brazil (Supplementary Figure 1) [20–47]. In our database, we reported the estimated F intake and urinary F monitoring. However, 19 publications only reported the estimated TDFI (Table 1) and the other nine reported both (Table 2). The 28 selected articles can be mentioned more than once depending on their categories of fluoridated tap water (explained in methods). Only two studies were performed with adults whose ages ranged from 20 to 35 years [28, 47]. Some studies present a high number of participants, however, in some of these publications, the number of children using dental care products can narrow down to 5% of the initial cohort [36]. In other publications, especially for urinary measures, the number of subjects is about 18 children. To simplify the figures, we compared the contribution of toothpaste only with dietary intake (such as water, beverages, and food sources) without taking into account supplements as only three studies mentioned them [20, 22, 41]. In most studies, the dental care product was toothpaste. Only one study had varnish in association with toothpaste [42]. F intake from the diet varied depending on the F concentration in tap water, meals, and beverages. It should be mentioned that local public water also affects the F content in meals (thus in diet) during the cooking. This additional F input was included into the diet.

In Figure 2, the TDFI (reported in μg/day in Figure 2A and μg/kg bw/day in Figure 2C) and the fraction of diet or toothpaste exposures were represented. Figures 2B,D showed the percentage of toothpaste in the total exposure.

Without acknowledging age or F concentration in water, total F intake was between 340 and 3,320 μg/day. Based on those concentrations, toothpaste F represented 15–95% of the TDFI reported in μg/day (Figures 2A,B); in the published percentages, its variation ranged from 19 to 84% (19 publications). This discrepancy is caused by the fact that the authors did not calculate all the toothpaste contribution percentages.

In Figure 2A, we can notice that the high F concentration in tap water was not associated with a higher input of the diet in the TDFI [21, 25, 27, 30, 42, 43, 45]. In Figures 2C,D, the age of the children was taken into consideration by dividing the body weight of the subjects. The TDFI was driven to the optimal range (50–70 μg/kg bw/day) or above due to the F intake from toothpaste (Figure 2C). The variations of F intake from diet seemed to be less sensitive to the F concentrations in water than the variations due to the concentrations and good practice of the use of dental care products in the area. The F intake from toothpaste represented 3–90% of the TDFI when reported to the body weight (Figure 2D), corresponding to 1–84% in published data, which discrepancy was due to the same reasons as those cited above.

Extremely high F concentrations in the water (>1.5 mg/L) were associated with a lower contribution of toothpaste, <20% of the total F intake. Among the three measurements included in our database, one was measured on an adult population supposed to have a better use of toothpaste (no swallowing) [47]. Therefore, between the two studies carried out in extremely high-fluoridated areas in children (>1.5 mg/L), only one reported an extremely high daily dietary input (Figure 2C) [36, 46].

When all the data were taken into consideration, the mean contribution of dental care products to the total exposure was 38 ± 27%. The F exposure through toothbrushing was thus significant when put into perspective with the TDFI for children: 39–51%, regardless of the F concentration in water (0.3–1.5 mg/L) [Table 3, the values reported by [20, 22, 24] were excluded]. However, in the case of extremely-fluoridated water (>1.5 mg/L), the dental care products contribution was estimated approximately 3% in the two studies carried out in children [36, 46].

**Table 3:** Contribution of dental care products in F exposure depending on the F concentration in tap water for children and adults.

**F in water**	**Concentration F (mg/L)**	**Mean fluoride from dental care products in the total exposure for children in % (sd %)**	**Mean fluoride from dental care products in the total exposure for adults in % (sd %)**
Poor	<0.3	45 (28) (*n* = 10)	12 (-) (*n* = 1)
Low	0.3–0.51	39 (24) (*n* = 7)	95 (-) (*n* = 1)
Medium	0.52–0.77	51 (24) (*n* = 14)	53 (39) (*n* = 2)
High	0.78–1.5	41 (24) (*n* = 8)	-
Extreme	>1.5	3 (2) (*n* = 2)	3 (-) (*n* = 1)

*The number of publications concerned by the category of F concentration in water was reported for each area*.

In adults, the mean contribution of dental care products to the total exposure was 12% in poor, 95% in low, 53% in medium, and 3% in extremely-fluoridated water (Table 3) [28, 47].

**Figure 3:**
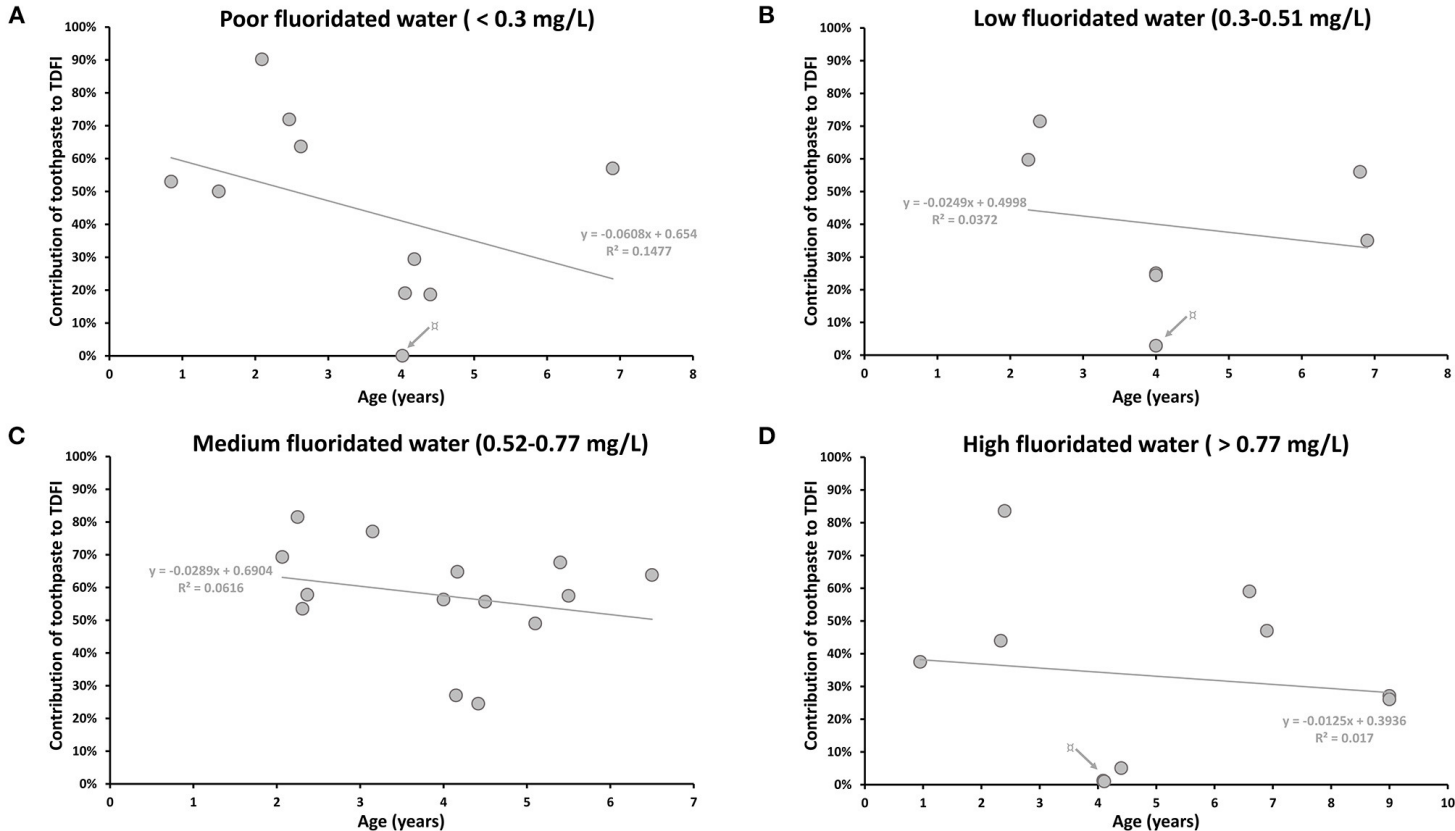
The contribution of daily toothpaste intake according to mean age (years) of participants in areas with different F concentration in drinking water (mg/L). **(A)** The contribution of toothpaste to the TDFI (%) in poor fluoridated water (<0.3 mg/L). ¤ Gray arrow indicates the lowest value from Abuhaloob et al. [36]: two toothpaste users among 81 participants. **(B)** The contribution of toothpaste to the TDFI (%) in low fluoridated water (0.3–0.51 mg/L). ¤ Gray arrow indicates the lowest value from Zohoori and Rugg-Gunn [40]: 3 toothpaste users among 28 in Darab (not the same region presented in Figure 2; Table 3). **(C)** The contribution of toothpaste to the TDFI (%) in medium fluoridated water (0.52–0.77 mg/L). **(D)** The contribution of toothpaste to the TDFI (%) in high fluoridated water (>0.77 mg/L). ¤ Gray arrow indicates the lowest values from Abuhaloob et al. [36]: nine toothpaste users among 135 participants.

The contribution of toothpaste in different fluoridated areas according to the mean age of participants was displayed in Figure 3. As most of the articles were kinetic studies, only values at peak-level were considered to evaluate the maximum effect of dental care products on the daily intake. Only data recorded 24 h after exposure have been reported for the kinetic studies. Data reported by Levy et al. [20, 22, 24] were not included because the different areas with different F concentrations could not be distinguished. Calculated correlations (*R*^2^) were all below 0.14 showing the absence of correlation between daily F intake from toothpaste and the age of children regardless of the tap water F concentrations (Figure 3).

Nevertheless, there was a tendency to present the highest estimation of daily F intake from toothpaste for the youngest children (younger than 4 years old) for all types of water (Figure 3). This observation was even more pronounced in poor-fluoridated areas, where the subjects under 4 years-old and older children were exposed to F from toothpaste between 50–90% and 0.04–57%, respectively (Figure 3A). The possible high contribution of toothpaste may be explained by the swallowing behavior for children under 4 years old [21, 25, 29, 30, 44].

Due to their better practice, adults should not be exposed to F through dental care products (Supplementary Figure 2) [28, 47]. However, Cardoso et al. (2006) reported high percentage values of toothpaste contribution that varied between 26 and 95% (Supplementary Figure 2) [28]. This high contribution for adult subjects was linked to their dental care practices. In this study, the authors actually reported that some subjects brushed their teeth three or four times a day. Another study also showed a non-negligible contribution of toothpaste to the TDFI, with an F ingestion from toothpaste of ~12 and 3% for 31 and 29 adults in poor and high-fluoridated areas, respectively [47].

**Figure 4:**
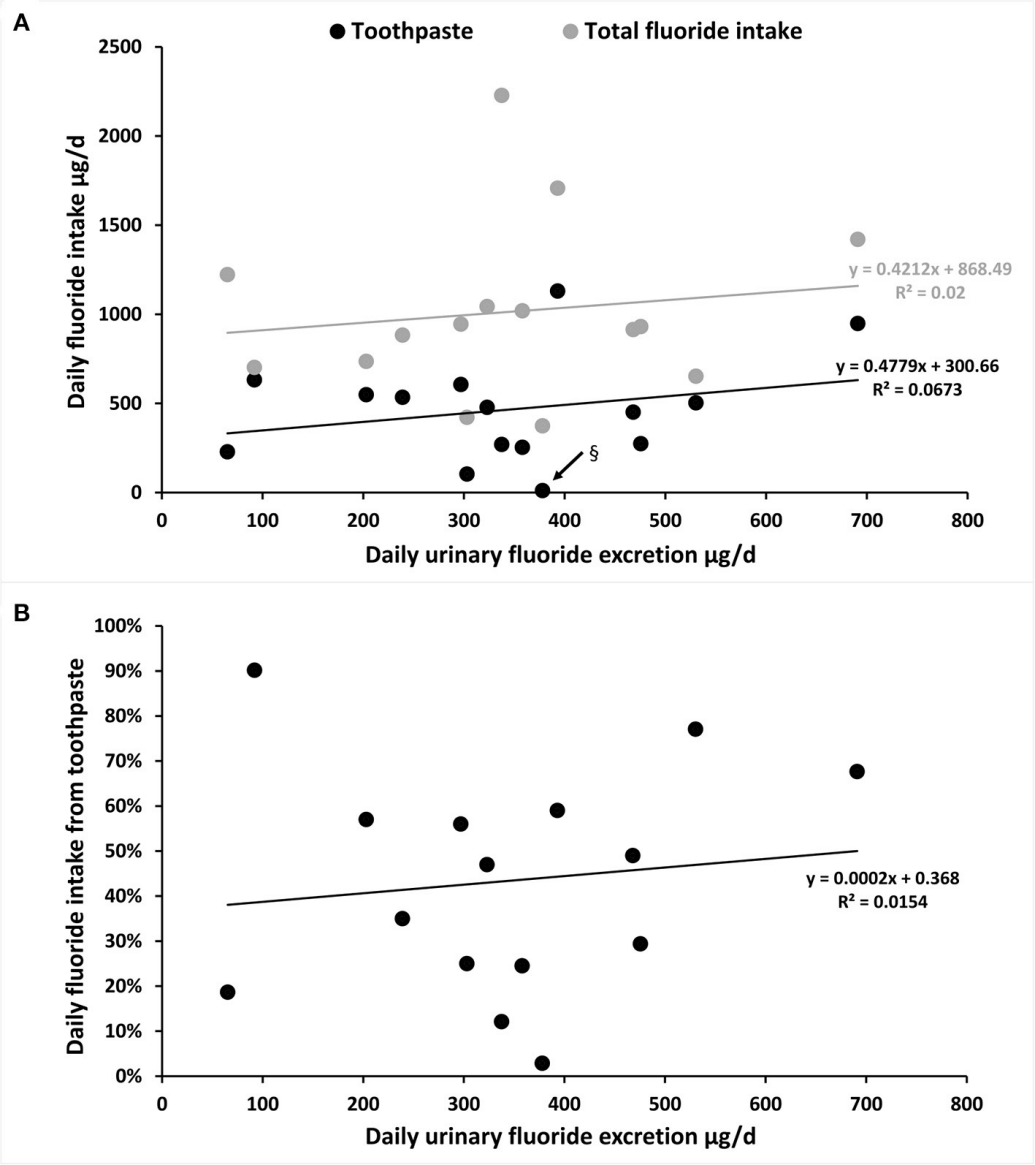
Estimation of the mean DUFE (μg/day) in relation with the mean TDFI (diet and toothpaste) or only daily F intake from toothpaste in participants aged 1–7 years old and 20–35 years old [the highest dot in **(A)**]. **(A)** The mean DUFE (μg/day) in relation with TDFI and daily F intake from toothpaste (μg/day). § Black arrow indicates the lowest value of F intake from toothpaste from Zohoori and Rugg-Gunn [40]: three toothpaste users among 28 in Darab (not the same region presented in Figure 2; Table 3). **(B)** The mean DUFE (μg/day) in relation with daily F intake from toothpaste (%).

To follow objectively the TDFI and to understand the capacity to eliminate absorbed F, we searched if there was a relation between the DUFE and the TDFI and the F intake from toothpaste (Figure 4A), as well as between the DUFE and the percentage of daily F intake from toothpaste (Figure 4B). For subjects living in high-fluoridated areas, data from Idowu et al. [46, 47] were removed.

There was a similar tendency to increase DUFE with increased TDFI and F intake through toothpaste (Figures 4A,B). However, with an *R*^2^ below 0.07, there was no correlation between DUFE and TDFI or between DUFE and daily F intake from toothpaste.

## Discussion

The present systematic review showed that the mean contribution of F-containing dental care products, mainly toothpastes, is 38% regardless of the age of children or F concentration in drinking water. These data are slightly lower than those published earlier by Paiva et al. [26], reporting a 65% contribution. The difference may be either due to the method of collecting data or to evolution of the use of less fluoridated toothpastes.

The contribution of F intake was not correlated with the age of children. However, children under 4 years old presented a very high TDFI as well as some adults who did not respect the good practice of dental care uses. Those two cases highlight the importance of: (i) dental products on the exposome, (ii) the types of F, bio-assimilation, and concentrations in the dental care products, and (iii) the importance of dental care products adapted to age, but more importantly, the results show the importance of good dental care habits (no swallowing, rinsing with water after toothbrushing, exposure to F after meals, and use of appropriate amount of toothpaste). The variation of F intake from the diet seemed to be less sensitive to the water concentration than the variation due to the concentration and good use of dental care products in the different areas. Therefore, despite the fact that studies in this field are lacking especially for adults, impact of dental care products on TDFI for adults should not be negligible. This requests to be further investigated in the case of misconducted use as it can increase the risk of excessive F intake.

The increase in F intake, especially due to dental care products did not necessarily correlate with the amount of F excretion. This result can be explained by the fact that we were looking at the reported means, which smoothed the values. The lack of correlation between DUFE and TDFI can also be due to (i) poor estimation of F inputs (additional sources and under or overestimation), and/or (ii) bias of the methodological and/or analytical quantification of urinary excretion (data were reported in means for each publication, choice of collection, and measure of the F into the urine), and/or (iii) variability of excretion capacity for each organism. In addition, these data were based on nine publications which could be a limitation of the study.

Urine is the only biomarker capable of measuring F excretion. However, urine may not be the most pertinent biomarker for the estimation of TDFI especially in children due to F accumulation during bone growth and mineralization. Children can retain more F in their skeleton (~50%) than adults (approximately 36%), with inverse retention in bone with age of the children and with the excess of F excreted in urine [67]. The absence of correlation between DUFE and TDFI suggests that there is a variability but a non-negligible amount of F was not eliminated from the organism. Our data showed a variation of DUFE between 65.2 and 691 μg/day that may have informed on the F bioavailability, its residence time (clearance), and its interactions with different tissues. The majority of body F is bound in hard tissues, such as bones and teeth, and <1% can be found in soft tissues [17].

Further investigation combining measures of F in plasma and urine could be informative on the bioavailability of F and its interactions with different organs. Once absorbed, F reaches peak serum concentrations after 20–60 min, and then returns to the baseline after approximately 15 h suggesting that part of the oral F passes through systemic route [56, 57, 68, 69]. This is probably the reason why a relation has been reported between supplement use or the amount of toothpaste used for brushing and child's fluorosis scores [65]. Most pharmacokinetic analyses showed a transient increase in the urinary F excretion approximately 1–3 h after topical application of fluoridated varnishes in adults and in children, after the use of a fluoridated mouthrinse solution, or after brushing with F-containing toothpastes [42, 56–58, 60, 64, 66]. A return to baseline is reported by all the studies 24–72 h after the end of the exposure, irrespective of the source. The minimal recommended period of urine collection is 24 h to obtain good estimations of the daily amount of F excretion. The DUFE is the variable generally recommended for the estimation of the daily F exposure. The amount of excreted F is obtained by multiplying the 24-h urinary volume by its F concentration [18].

As a consequence, we have proposed an experimental model of cumulative F exposure following the age of the individuals considering three different thresholds of 30, 300, and 1,300 μg F/day (Supplementary Figure 3) and a model of mixed exposures (1,300 μg F/day until 4 years, then 300 μg F/day until 8 years, and 30 μg F/day until 16 years). The thresholds have been defined based on the estimated F retention. Those values were obtained by subtracting the DUFE from the TDFI and were estimated between a few μg and 1,890 μg/day. Thus, this model is a cumulative representation, which includes daily F bone retention to estimate the trapped F into the body over a span of several years. According to this model, early age exposure could drastically affect the total F retention into the organism. Even though the residence time (i.e., half-life) of F into the different organs remains not well known, the exposure to a high absorption of dental care products may print a high F content over the years. In addition, bad habits of dental care products may impact F exposure for the adults. Therefore, these data showing a non-negligible contribution to daily F intake through toothbrushing using F-containing toothpastes may be discussed in the light of the literature as F was reported to pass through the blood-placental barrier and the blood-brain barrier thus subsequently cause learning problems [70]. In fact, most of studies on the safety of toothpastes and dental care products are short-term pharmacokinetics studies that do not consider long-term effects, whereas F accumulated in bone may be released in specific situations associated to skeletal loss, such as lactation [71]. However, we found no study that has explored the contribution of dental care products to total F exposure in pregnant and lactating women nor studies taking into account the gender, especially in young children. This concern is even more important considering that recent studies reported a relation between prenatal F exposure and lower performance intelligence quotient (IQ) in boys, but not in girls [72]. An increase of 0.5 mg/L of F concentration in the water (approximately equal to the difference between fluoridated and non-fluoridated regions) was associated with a 7.9-point lower IQ score in formula-fed infants and 6.3-point lower IQ score in breastfed children in both boys and girls, suggesting that postnatal exposure to F may affect both sexes [73]. Sex-dependent susceptibility to F may be due to multiple biological and behavioral reasons, they have also been reported in several experimental studies in rodents, and more recently in zebrafish [12, 13].

In conclusion, our review highlights the major F contribution from dental care products regardless of the area or F concentration in drinking water. This additional source presents a large variability depending on the concentration, chemical forms, and amount of the dental product used. However, the good usage of these products also seems to be determinant for the contribution to TDFI. Therefore, the contribution of F intake through toothpaste can be easily controlled and adapted to the patient. Consequently, the future studies on F exposure and toxicity need to take into consideration exposure to F-containing dental care products, habits of use, and individual features (gender, age, diet, caries, etc.). Furthermore, considering the contribution of dental care products to the TDFI, the “optimal daily F intake” estimated approximately 50–70 μg/kg bw/day by EFSA could be reevaluated to determinate the optimal DDFI depending on each individual. The contribution of ~39–51% due to dental care products suggests that the optimal daily dietary F may be half of the EFSA values.

## Data Availability Statement

The original contributions presented in the study are included in the article/Supplementary Material, further inquiries can be directed to the corresponding author.

## Author Contributions

HS and SH selected the papers and reviewed them independently. SB was the third reviewer. HS and RE organized the data. HS, SH, and SB analyzed the data and the discussion. SH and SB drafted the manuscript. All authors contributed to the writing of the article and approved the final version.

## Funding

The study was funded by the French National Institute of Health and Medical Research (INSERM), the Université Paris Cité (Idex Project FLUOREMAIL), and the National Agency for Safety of Food and Environment (ANSES) (Grant 2019/1/230).

## Conflict of Interest

The authors declare that the research was conducted in the absence of any commercial or financial relationships that could be construed as a potential conflict of interest.

## Publisher's Note

All claims expressed in this article are solely those of the authors and do not necessarily represent those of their affiliated organizations, or those of the publisher, the editors and the reviewers. Any product that may be evaluated in this article, or claim that may be made by its manufacturer, is not guaranteed or endorsed by the publisher.
