# Supplementary material for: Fluoride Intake Through Dental Care Products: A Systematic Review
Source: Front Oral Health. 2022 Jun 10;3:916372. doi: 10.3389/froh.2022.916372 (PMC9231728; doi:10.3389/froh.2022.916372)
Supplement: Supplementary file 1 [file Data_Sheet_1.docx]

**Supplementary data**

**This document contains:**

**3 figures**


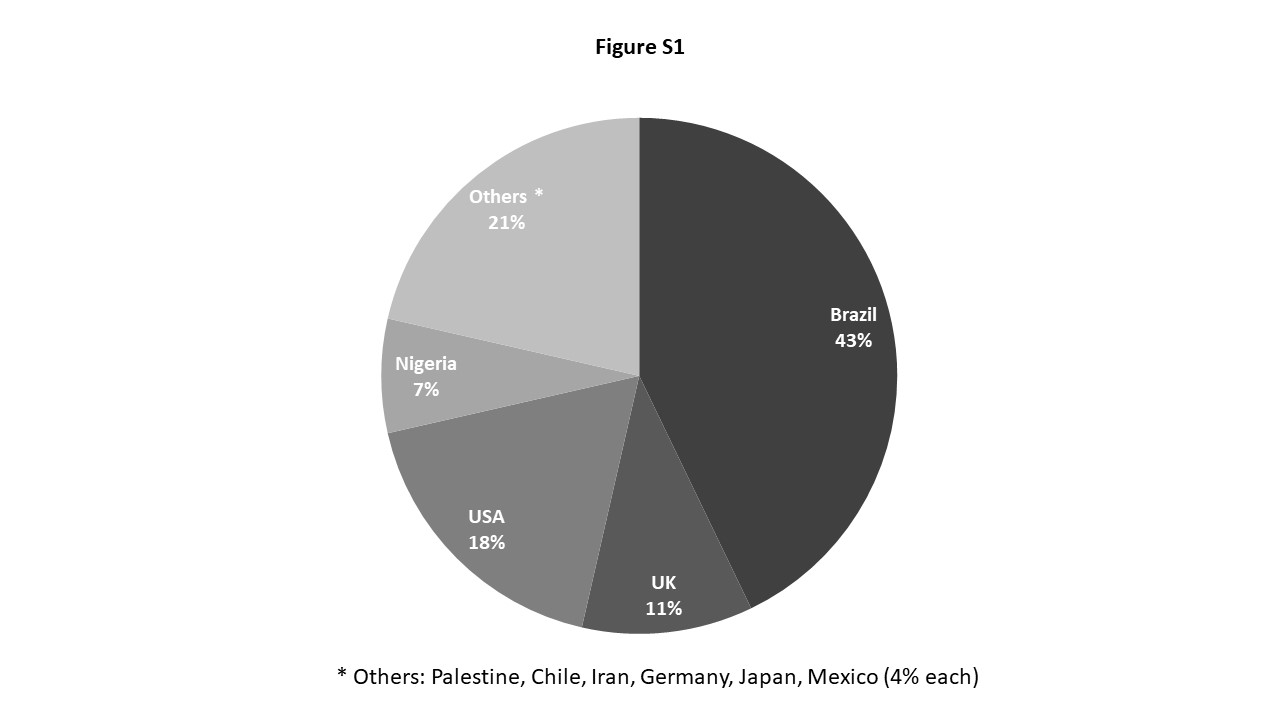
 **Figure S1.** Mondial geographical repartition of the included studies.

*
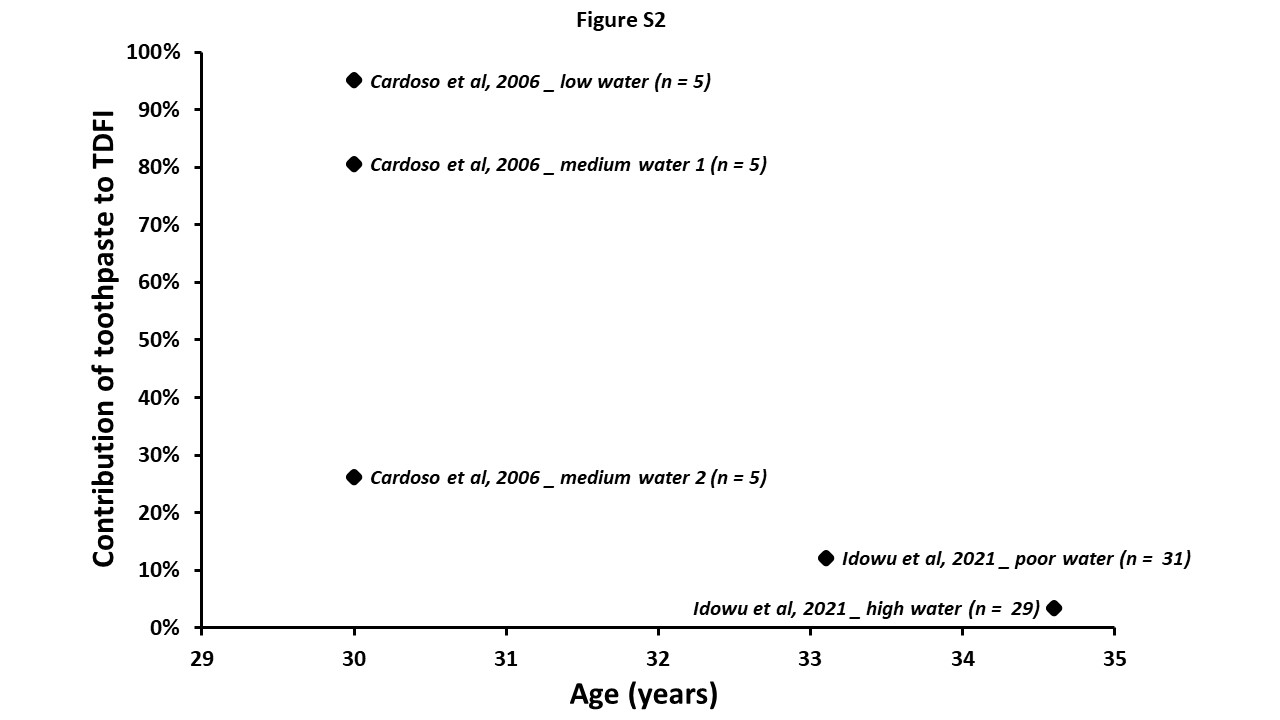
***Figure S2. Contribution of toothpaste to the TDFI (%) in adults.** Only 2 articles are related to adults in our PRISMA selection.


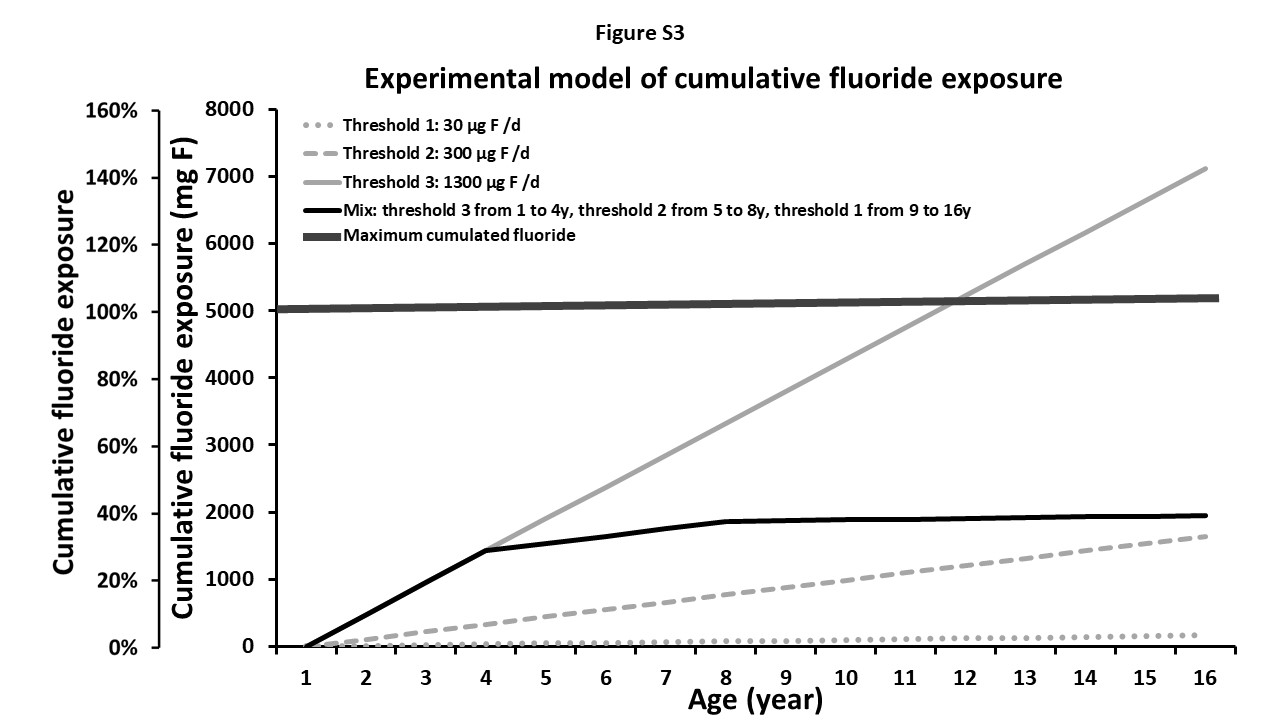
**Figure S3. Experimental model of cumulative fluoride exposure following the age of the individuals.** It considers 3 different thresholds of 30, 300 and 1300 µg F/d and a model of mixed exposures (1300 µg F/d until 4 years, then 300 µg F/d until 8 years and 30 µg F/d until 16 years). The maximum cumulated fluoride exposure (100 %) is reached at 5000 mg.
